# Hsa_circ_0048179 attenuates free fatty acid-induced steatosis via hsa_circ_0048179/miR-188-3p/GPX4 signaling

**DOI:** 10.18632/aging.104081

**Published:** 2020-11-18

**Authors:** Wenjun Yang, Jinduo Zhao, Ye Zhao, Wenfeng Li, Liang Zhao, Yi Ren, Rongying Ou, Yunsheng Xu

**Affiliations:** 1 Department of Hepatobiliary Surgery, The First Affiliated Hospital of Wenzhou Medical University, Wenzhou, Zhejiang, China; 2 Department of Gynaecology and Obstetrics, The First Affiliated Hospital of Wenzhou Medical University, Wenzhou, Zhejiang, China; 3 Laboratory for Advanced Interdisciplinary Research, Institutes of Translational Medicine, The First Affiliated Hospital of Wenzhou Medical University, Wenzhou, Zhejiang, China; 4 Department of Biomedical Sciences, Florida State University College of Medicine, Tallahassee, FL 32304, USA; 5 Department of Dermatovenereology, The Seventh Affiliated Hospital of Sun Yat-sen University, Shenzhen, Guangdong, China

**Keywords:** non-alcoholic fatty liver disease, lipid accumulation, hsa_circ_0048179, GPX4, miR-188-3p

## Abstract

Although circular RNAs (circRNAs) are known to play key roles in non-alcoholic fatty liver disease, much about their targets and mechanisms remains unknown. We therefore investigated the actions and mechanisms of hsa_circ_0048179 in an *in vitro* model of NAFLD. HepG2 cells were exposed to oleate/palmitate (2:1 ratio) for 24 h to induce intracellular lipid accumulation. Using CCK-8 assays, flow cytometry, fluorescence microscopy, western blotting, RT-qPCR, and Oil red O staining, we found that oleate/palmitate treatment reduced cell viability while increasing apoptosis and lipid accumulation in HepG2 cells. Levels of the antioxidant enzyme GPX4 were decreased in oleate/palmitate-treated HepG2 cells, and there were corresponding increases in reactive oxygen species and damage to mitochondrial cristae. Levels of hsa_circ_0048179 expression were also suppressed by oleate/palmitate treatment, and GPX4 levels were markedly increased in HepG2 cells following transfection with hsa_circ_0048179. Analysis of its mechanism revealed that hsa_circ_0048179 upregulated GPX4 levels by acting as a competitive “sponge” of miR-188-3p and that hsa_circ_0048179 attenuated oleate/palmitate-induced lipid accumulation in HepG2 cells by sponging miR-188-3p. Collectively, our findings suggest that hsa_circ_0048179 may play a key role in the pathogenesis of steatosis and may thus be a useful target for drug development.

## INTRODUCTION

The defining characteristic of non-alcoholic fatty liver disease (NAFLD), which is the most commonly occurring ailment affecting that organ [1], is excessive deposition of triglycerides within the liver tissue in the absence of alcohol consumption [2]. The main NAFLD subgroup is non-alcoholic steatohepatitis (NASH) [3]. The earliest stage of NAFLD is hepatic steatosis, which is often self-limited [4], but in some cases progresses to NASH and, ultimately, to fibrosis and cirrhosis [5]. NAFLD is characterized by lipid accumulation within liver tissue not due to alcohol abuse [6, 7]. NAFLD severely affects the quality of life of patients and is a financial burden for society [8, 9]. However, the pathogenesis of NAFLD has not been entirely elucidated. It is therefore important to continue to investigate the pathogenesis of NAFLD and expand the available treatment options.

Circular RNA (circRNA) is an endogenous noncoding RNA molecule ubiquitous in eukaryotes [10]. CircRNAs have a closed loop structure with no 5’ cap or 3’ poly(A) tail, and often originate from exons or introns within gene fragments [10, 11]. These molecules are now known to play vital roles in a number of cellular processes, including cell growth, migration, and invasion, as well as carcinogenic transformation [12]. CircRNAs regulate gene expression by binding to proteins or sponging microRNAs (miRNAs) [13]. Recently, Jin et al. reported that circRNAs may function as molecular diagnostic biomarkers of NASH acting via a circRNA-miRNA-mRNA axis [14]. For example, circRNA_0046366 could inhibit hepatocellular steatosis in high fat-treated HepG2 cells via miR-34a/PPARα axis [15]. In addition, hsa_circ_0048179 was found to be expressed in the HepG2 human hepatoblastoma cell line, normal human epidermal keratinocytes (NHEK), and HeLa-S3 cell line [16]. However, the potential role of hsa_circ_0048179 in the progression of NAFLD and its underlying mechanism remain unclear. In the present study, therefore, we explored the roles of hsa_circ_0048179 in the pathogenesis of NAFLD.

## RESULTS

### *In vitro* oleate/palmitate-induced NAFLD model

**Figure 1:**
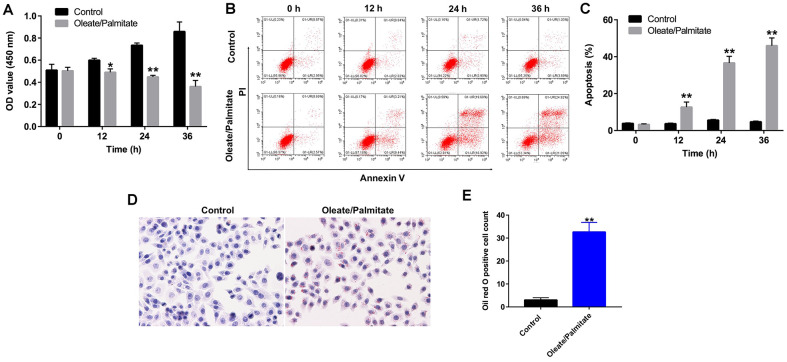
**Oleate/palmitate induces an *in vitro* model NAFLD.** HepG2 cells were incubated with oleate/palmitate (2:1 molar ratio) for 0, 12, 24 and 36 h. (**A**) CCK-8 assays were used to measure cell viability. (**B**, **C**) Apoptotic cells were detected with Annexin V and PI double staining. (**D**, **E**) Lipid deposition in HepG2 cells detected with Oil red O staining. Magnification: 200x. *P<0.05, **P<0.01 vs. control group.

HepG2 cells were exposed to oleate/palmitate for 24 h to induce intracellular lipid accumulation. CCK-8 assays were used to assess the viability of HepG2 cells after treatment with oleate/palmitate for 12, 24 and 36 h. Incubation in the presence of oleate/palmitate led to significant time-dependent decreases in HepG2 cell viability and corresponding increases in apoptosis (Figure 1A). In addition, apoptosis rate was 12.65 ± 2.78%, 36.60 ± 3.61% and 45.97 ± 4.21% in HepG2 cells after treatment with oleate/palmitate for 12, 24 and 36 h respectively, compared with control (3.26 ± 0.35%) (Figure 1B, 1C). Oil red O staining revealed that intracellular accumulation of lipid was significantly enhanced in oleate/palmitate-treated HepG2 cells (Figure 1D, 1E). These data illustrate that oleate/palmitate induces the apoptosis and lipid accumulation characteristic of NAFLD in HepG2 cells. We have thus established an *in vitro* model of NAFLD. To maximize fat accumulation while minimizing cytotoxicity, incubation with oleate/palmitate for 24 h was selected for subsequent experiments.

### Oleate/palmitate induces ROS generation and decreases mitochondrial membrane potentials (MMPs) in HepG2 cells

**Figure 2:**
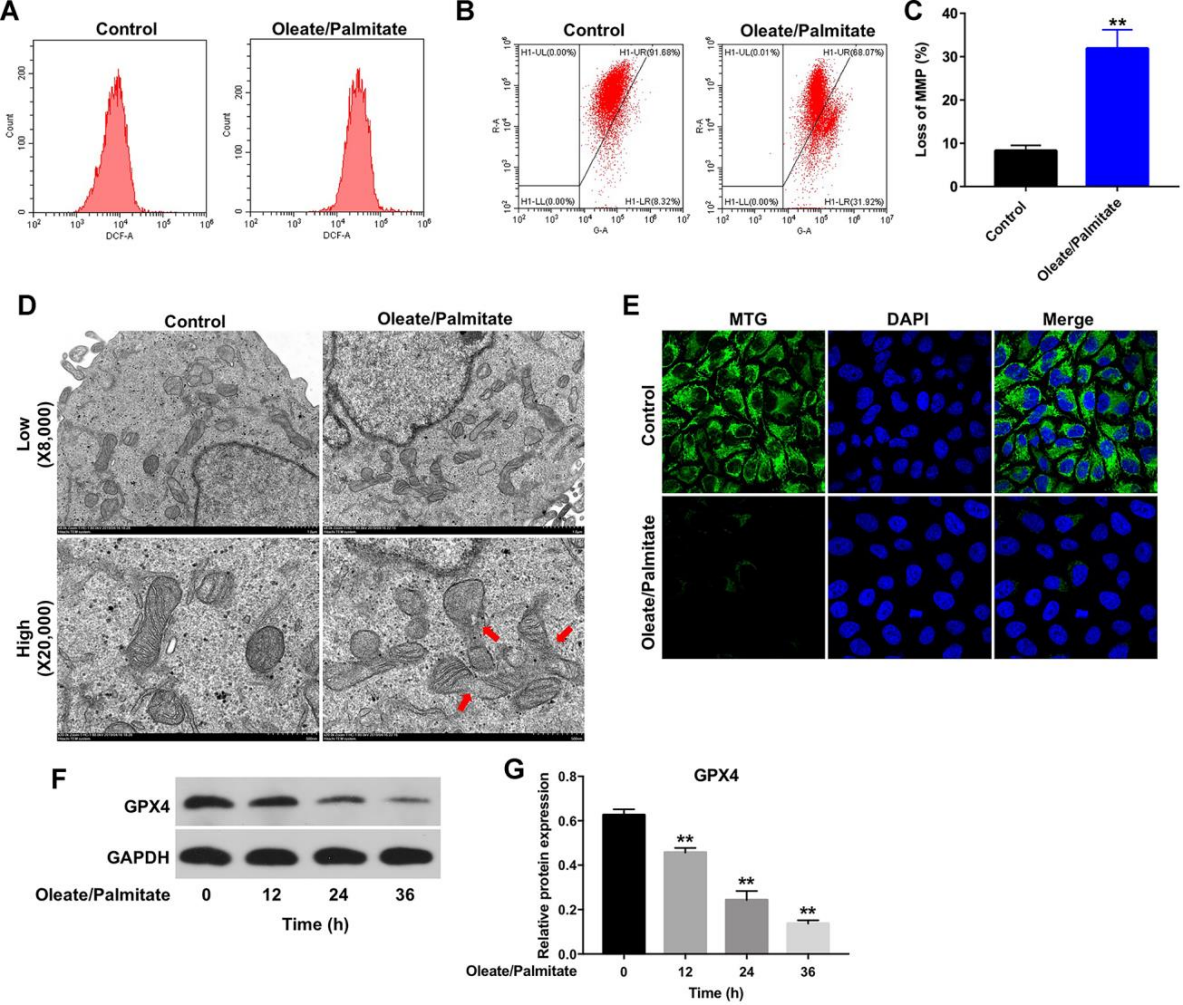
**Oleate/palmitate induces ROS generation and decreases MMP in HepG2 cells.** HepG2 cells were incubated with oleate/palmitate (2:1 molar ratio) for 24 h. (**A**) ROS generation was detected as DCF fluorescence using flow cytometry. (**B**, **C**) JC-1 staining was used to detect mitochondrial membrane depolarization. (**D**) Changes in mitochondrial morphology were observed in HepG2 cells using TEM. The mitochondria from oleate/palmitate-treated HepG2 cells were swollen and cristae appeared disrupted (red arrows). Magnification: 20,000x. (**E**) Mitochondrial distribution in HepG2 cells detected with MTG staining. The green color represents mitochondria staining. (**F**) HepG2 cells were incubated with oleate/palmitate (2:1 molar ratio) for 0, 12, 24 and 36 h. Levels of GPX4 expression in HepG2 cells were detected with western blotting. GAPDH was used as an internal control. (**G**) Relative expression of GPX4 in HepG2 cells was quantified by normalization to β-actin. **P<0.01 vs. control group.

It was previously reported that lipid deposition in hepatocytes leads to increased ROS generation and mitochondrial dysfunction [17]. As shown in Figure 2A, oleate/palmitate markedly increased ROS generation in HepG2 cells, as indicated by the increased DCFH-DA fluorescence intensity. In addition, oleate/palmitate exposure also significantly decreased MMPs in HepG2 cells (Figure 2B, 2C), and transmission electron microscopy (TEM) revealed mitochondrial swelling and fragmentation of the mitochondrial cristae in the oleate/palmitate-treated cells (Figure 2D). Moreover, MTG staining results indicated that oleate/palmitate exposure decreased the mitochondrial fluorescence intensity in HepG2 cells, compared with control group (Figure 2E). Glutathione peroxidase 4 (GPX4), a phospholipid hydroperoxidase, protects cells against membrane lipid peroxidation [18]. As shown in Figure 2F, 2G, oleate/palmitate exposure resulted in decreased expression of GPX4 in HepG2 cells in a time-dependent manner.

### Hsa_circ_0048179 increases GPX4 levels in HepG2 cells

**Figure 3:**
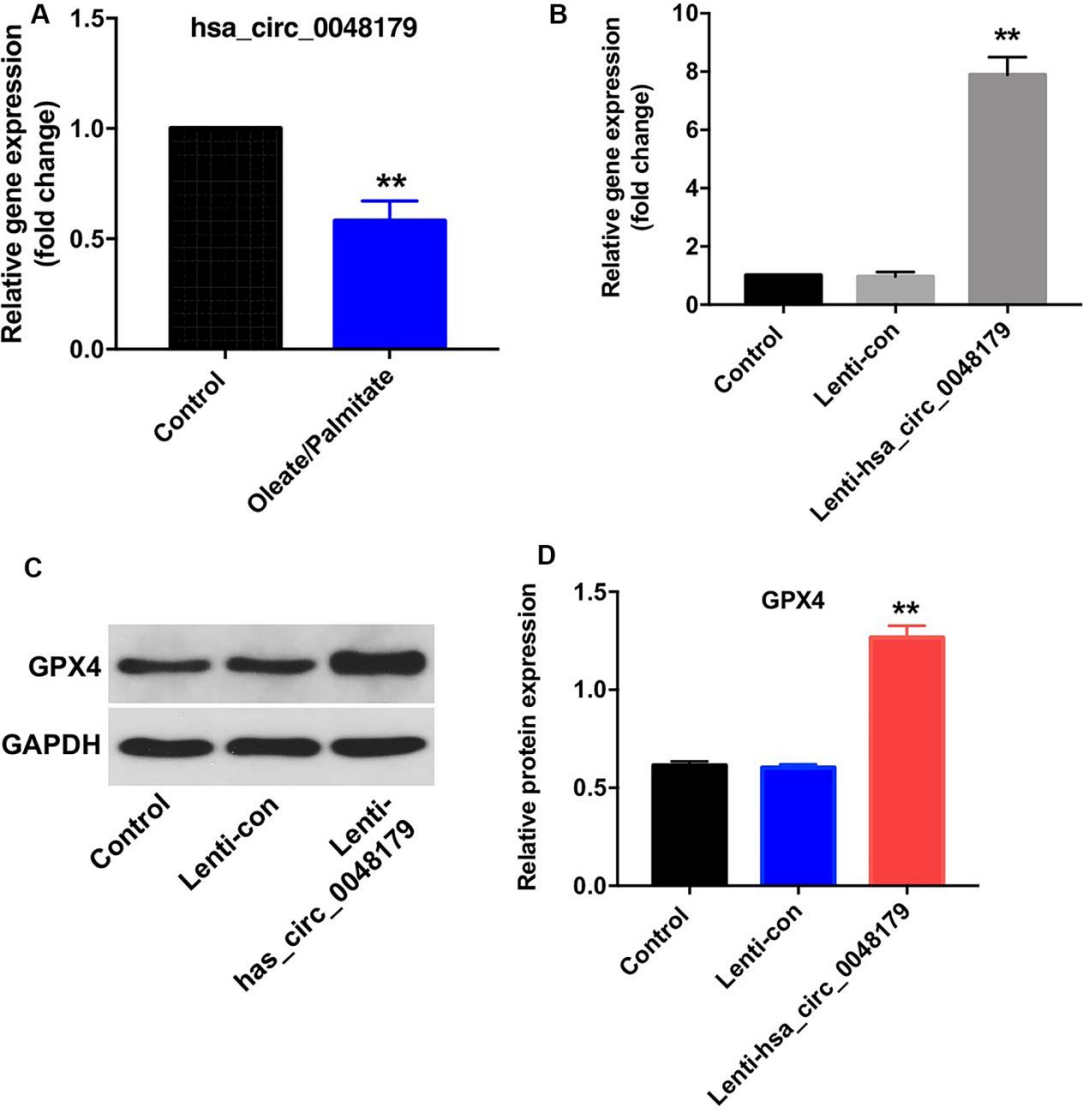
**Overexpression of hsa_circ_0048179 increases GPX4 levels in HepG2 cells.** (**A**) HepG2 cells were incubated with oleate/palmitate (2:1 molar ratio) for 24 h, after which levels of hsa_circ_0048179 were detected using qRT-PCR. (**B**) HepG2 cells were transfected with NC or lenti-hsa_circ_0048179 for 72 h. Hsa_circ_0048179 levels in HepG2 cells were detected using qRT-PCR. (**C**) Levels of GPX4 expression in HepG2 cells were detected with western blotting. (**D**) Relative expression of GPX4 in HepG2 cells was quantified by normalization to GAPDH. **P<0.01 vs. control group.

Has_circRNA_0048179 is reported to be expressed in HepG2 cells [16]. As shown in Figure 3A, hsa_circ_0048179 levels were significantly lower in oleate/palmitate-treated HepG2 cells than in untreated control cells. To determine the function of hsa_circ_0048179, we used a lentiviral vector to overexpress it HepG2 cells (Figure 3B). We found that levels of GPX4 were upregulated in cells overexpressing hsa_circ_0048179 (Figure 3C, 3D). Moreover, overexpression of hsa_circ_0048179 led to increased expression of GPX4 in oleate/palmitate-treated HepG2 cells in a time-dependent manner (Supplementary Figure 1A, 1B). These data suggest that hsa_circ_0048179 may suppress lipid deposition and mitochondrial dysfunction by mediating enhancement of GPX4 activity.

### Hsa_circ_0048179 functions as a ceRNA targeting miR-188-3p in NAFLD

**Figure 4:**
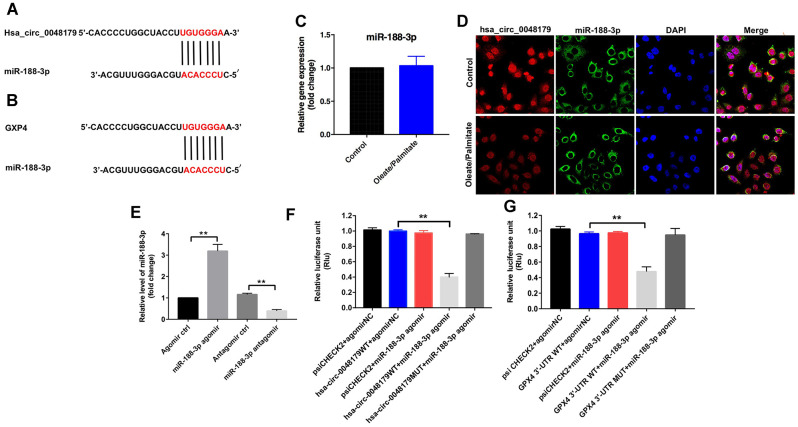
**miR-188-3p forms a bridge connecting hsa_circ_0048179 and GPX4.** (**A**) Sequence alignment of miR-188-3p with the putative binding sites within the WT regions of hsa_circ_0048179. (**B**) Sequence alignment of miR-188-3p with the putative binding sites within the WT regions of GPX4. (**C**) HepG2 cells were incubated with oleate/palmitate (2:1 molar ratio) for 24 h. after which levels of miR-188-3p expression were detected using qRT-PCR. (**D**) Cellular localization of hsa_circ_0048179 and miR-188-3p in HepG2 cells were analyzed using FISH assays. (**E**) HepG2 cells were transfected with miR-188-3p agomir or miR-188-3p antagomir for 72 h, after which levels of miR-188-3p expression were detected using qRT-PCR. **P<0.01 (**F**) Dual luciferase reporter assays were used to detect the luciferase activity in 293T cells following co-transfection with hsa_circ_0048179-WT/MUT 3’-UTR plasmid and miR-188-3p agomir. **P<0.01 (**G**) Dual luciferase reporter assays were used to detect luciferase activity in 293T cells after co-transfection of GPX4-WT/MUT 3′-UTR plasmid and miR-188-3p agomir. **P<0.01.

CircRNAs regulate gene expression by binding to proteins or sponging miRNA [19]. Data from the online bioinformatics tool circbase (http://www.circbase.org) and the circular RNA interactome (https://circinteractome.nia.nih.gov/index.html) indicate that hsa_circ_0048179 is an exonic circRNA, which are known to regulate expression of target genes by sponging their miRNAs [13]. We therefore used the Circular RNA interactome, TargetScan (http://www.targetscan.org/vert_71/) and miRDB (http://www.mirdb.org) to identify miRNAs that interact with hsa_circ_0048179 and target GPX4. The bioinformatics results predicted that miR-188-3p was a likely target of both hsa_circ_0048179 and GPX4 (Figure 4A, 4B). Moreover, levels of miR-188-3p were unaffected by oleate/palmitate stimulation of HepG2 cells (Figure 4C), and fluorescence in situ hybridization (FISH) assays showed that hsa_circ_0048179 and miR-188-3p partially co-localized in the cytoplasm (Figure 4D). This suggests that hsa_circ_0048179 directly interacts with miR-188-3p and that hsa_circ_0048179 functions as a ceRNA targeting miR-188-3p in NAFLD.

qRT-PCR results indicated that miR-188-3p levels were markedly increased in HepG2 cells following transfection with miR-188-3p agomir, and significantly decreased following transfection with miR-188-3p antagomir (Figure 4E). Furthermore, dual-luciferase reporter assays demonstrated that miR-188-3p inhibited hsa_circ_0048179-WT, while hsa_circ_0048179-MT was unaffected (Figure 4F). This suggests miR-188-3p is a potential binding target of hsa_circ_0048179. Luciferase assays also showed that miR-188-3p inhibits expression of GPX4-WT by not GPX4-MT (Figure 4G), indicating that GPX4 mRNA is a potential binding target of miR-188-3p. Thus hsa_circ_0048179 may upregulate GPX4 levels by acting as a competitive “sponge” of miR-188-3p.

### Downregulation of miR-188-3p attenuates oleate/palmitate-induced lipid accumulation in HepG2 cells

**Figure 5:**
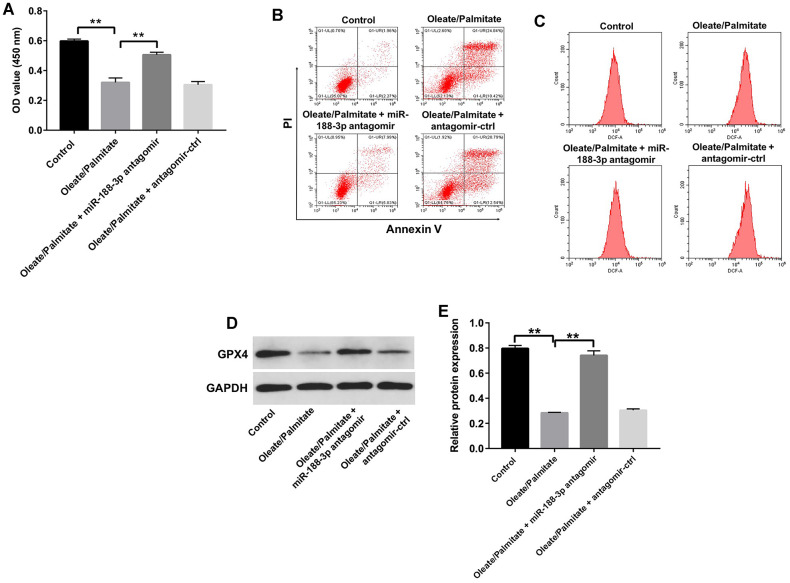
**Downregulation of miR-188-3p attenuates oleate/palmitate-induced lipid accumulation in HepG2 cells.** (**A**) HepG2 cells were transfected with miR-188-3p antagomir for 48 h and then exposed to oleate/palmitate (2:1 molar ratio) for another 24 h. CCK-8 assays were used to measure cell viability. **P<0.01 (**B**) Apoptotic cells were detected with Annexin V and PI double staining. (**C**) ROS generation were detected as DCF fluorescence with flow cytometry. (**D**) Levels of GPX4 expression in HepG2 cells were detected with western blotting. (**E**) Relative GPX4 expression in HepG2 cells was quantified by normalization to GAPDH. **P<0.01.

Using CCK-8 assays and flow cytometry, we next assessed the effect of miR-188-3p on the viability HepG2 cells. Suppression of miR-188-3p using miR-188-3p antagomir markedly reversed oleate/palmitate-induced cytotoxicity and apoptosis and diminished ROS generation in HepG2 cells (Figure 5A–5C). In addition, transfection with miR-188-3p antagomir also reversed oleate/palmitate-induced suppression of GPX4 expression (Figure 5D, 5E). These results suggest that downregulation of miR-188-3p may attenuate oleate/palmitate-induced steatosis.

### Overexpression of hsa_circ_0048179 attenuates oleate/palmitate-induced lipid accumulation in HepG2 cells by sponging miR-188-3p

**Figure 6:**
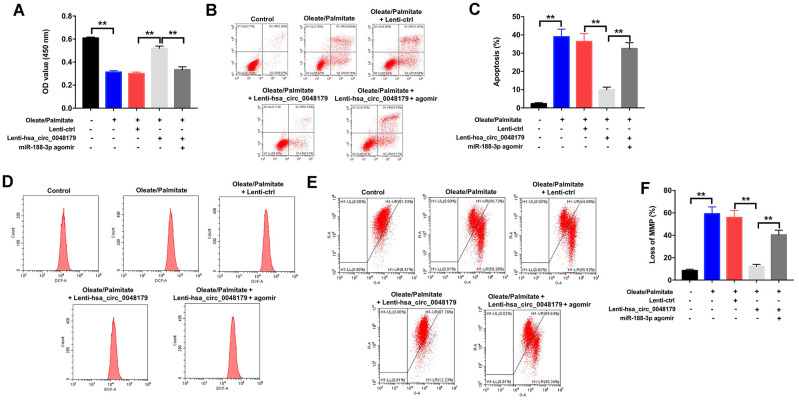
**Overexpression of hsa_circ_0048179 attenuates oleate/palmitate-induced lipid accumulation in HepG2 cells by sponging miR-188-3p.** HepG2 cells were transfected with miR-188-3p agomir or co-transfected with hsa_circ_0048179 and miR-188-3p agomir for 48 h, and then exposed to oleate/palmitate (2:1 molar ratio) for another 24 h. (**A**) CCK-8 assays were used to measure cell viability. **P<0.01 (**B**, **C**) Apoptotic cells were detected with Annexin V and PI double staining. **P<0.01 (**D**) ROS generation were detected as DCF fluorescence with flow cytometry. (**E**, **F**) JC-1 staining was used to determine mitochondrial membrane depolarization. **P<0.01.

Having verified that hsa_circ_0048179 targets miR-188-3p, the mechanism of action of hsa_circ_0048179 in oleate/palmitate-induced lipid accumulation in HepG2 cells was still unknown. Oleate/palmitate significantly inhibited HepG2 cell viability while increasing both apoptosis and ROS levels within the cells, but these effects were reversed by infection with lenti-hsa_circ_0048179 viral vector. However, when HepG2 cells were treated with hsa_circ_0048179 plus miR-188-3p agomir in the presence of oleate/palmitate, the favorable effects of hsa_circ_0048179 were suppressed by the miR-188-3p overexpression (Figure 6A–6D). As summarized above, oleate/palmitate significantly decreased MMPs in HepG2 cells. This effect too was reversed by transfection with lenti-hsa_circ_0048179, and again the beneficial effect of hsa_circ_0048179 was suppressed by miR-188-3p agomir (Figure 6E, 6F). These results further illustrate that overexpressed hsa_circ_0048179 attenuates oleate/palmitate-induced lipid accumulation in HepG2 cells by sponging miR-188-3p.

## DISCUSSION

**Figure 7:**
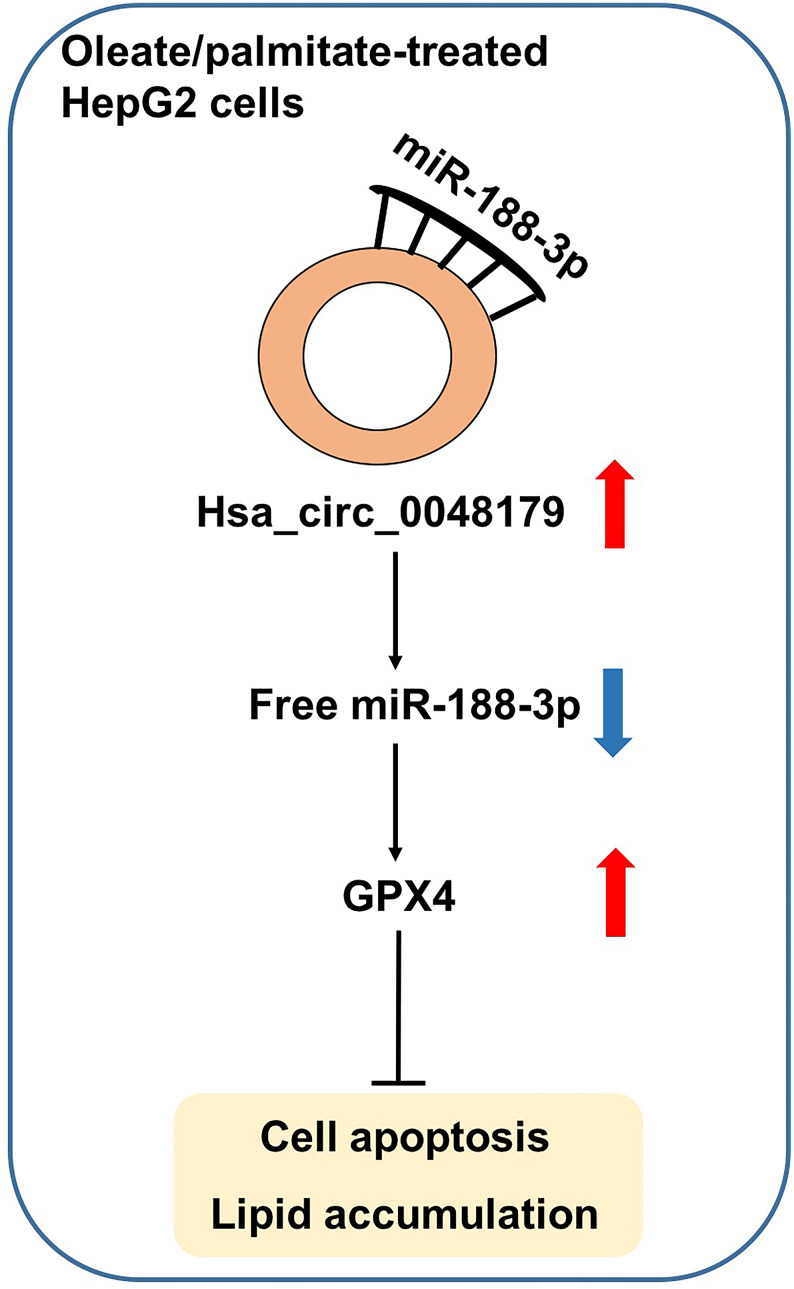
**Hsa_circ_0048179 attenuated this oleate/palmitate-induced steatosis in HepG2 cells via hsa_circ_0048179/miR-188-3p/GPX4 signaling.** Hsa_circ_0048179 attenuated oleate/palmitate-induced steatosis in HepG2 cells through upregulating GPX4 partially via ‘sponging’ miR-188-5p.

CircRNAs are important epigenetic regulators of miRNA-mRNA interactions [15]. In the present study, we observed that hsa_circ_0048179 levels were significantly decreased in HepG2 cells made steatotic through exposure to oleate/palmitate fatty acids. Overexpression of hsa_circ_0048179 attenuated this oleate/palmitate-induced steatosis via hsa_circ_0048179/miR-188-3p/GPX4 signaling (Figure 7).

The hallmark in NASH pathogenesis is lipid deposition within hepatocytes [17]. Ricchi et al. found that oleic and palmitic acids most effectively induced steatotic deposition in hepatocyte cultures [20]. It has been shown that apoptosis and ROS generation are associated with the progression of NASH, and overproduction of ROS may lead to mitochondrial oxidative stress [21, 22]. ROS, including O2·^-^, H_2_O_2_ and ONOO^−^, are produced in all types of cells [23]. In hepatocytes, O2·^-^ is normally converted to H_2_O_2_ and then to H_2_O by antioxidant GPX enzymes [24]. GPX4 is able to directly reduce fatty acid and phospholipid hydroperoxide [25]. In the present study, oleate/palmitate-induced fatty acid accumulation was accompanied by significant reductions in GPX4 levels, which likely account for the increased ROS levels and reduced MMPs.

CircRNAs are reported to be differentially expressed in HepG2 cells, as compared to non-cancer cells [16]. Our study is the first to directly investigate the association between hsa_circ_0048179 levels and steatosis. We found that hsa_circ_0048179 levels are downregulated in oleate/palmitate-treated HepG2 cells and that GPX4 expression was markedly increased in HepG2 cells infected with lenti-hsa_circ_0048179 viral vector. Because exon-derived circRNAs reportedly regulate target genes by sponging miRNAs [13], we used the Circular RNA interactome, Targetscan and miRDB to identify miRNAs interacting with hsa_circ_0048179 and the GPX4 3’-UTR. We found miR-188-3p to be a potential common target for both hsa_circ_0048179 and GPX4. Consistent with that finding, dual-luciferase reporter and FISH assays indicated that miR-188-3p is the binding target of hsa_circ_0048179, while GPX4 is the target of miR-188-3p. Taken together, these findings suggest NAFLD may be related to abnormalities in hsa_circ_0048179/miR-188-3p/GPX4 signaling.

Although our findings implicate hsa_circ_0048179 in steatosis, the mechanism by which it affects lipid accumulation in oleate/palmitate-treated HepG2 cells remains unclear. We found that downregulation of miR-188-3p attenuated oleate/palmitate-induced apoptosis, ROS generation and mitochondrial damage in HepG2 cells, while overexpression of hsa_circ_0048179 reversed those adverse effects. Moreover, the beneficial effects of overexpressed hsa_circ_0048179 were, in turn, reversed by transfection of miR-188-3p agomir. These results demonstrate that overexpression of hsa_circ_0048179 attenuates oleate/palmitate-induced lipid accumulation in HepG2 cells by sponging miR-188-3p and that the effects of hsa_circ_0048179 are attributable, at least in part, to its function as a ceRNA targeting miR-188-3p.

## MATERIALS AND METHODS

### Cell culture and treatment

HepG2 human hepatoblastoma cells were purchased from the American Type Culture Collection (ATCC, Rockville, MD, USA) and were maintained in Dulbecco's modified Eagle's medium (DMEM; Thermo Fisher Scientific, Waltham, MA, USA) supplemented with 10% fetal bovine serum (FBS; Thermo Fisher Scientific) and 100 units/ml penicillin/streptomycin at 37° C and 5% CO_2_. For oleate/palmitate treatment, the HepG2 cells were incubated with free fatty acids (oleate/palmitate, 2:1 molar ratio, to achieve a final concentration of 1 mM fatty acids) for 24 h, and total RNA and protein were extracted for experimentation [15, 26].

### Lentivirus production and cell transfection

Has_circ_004817 was synthesized by GenePharma (Shanghai, China) and subcloned into the pHBLV-ciR vector (HANABIO, Shanghai, China). Lentivirus carrying has_circ_004817 was packaged in 293T cells, after which the supernatant was concentrated. HepG2 cells were then infected for 72 h using the lenti-has_circ_004817 supernatant.

Agomir NC, miR-188-3p agomir, antagomir NC and miR-188-3p antagomir were purchased from GenePharma and respectively transfected into HepG2 cells using Lipofectamine 2000 (Thermo Fisher Scientific) according to the manufacturer’s instructions. The culture medium containing 10% FBS was changed after 6 h of transfection, and the cells were then incubated for additional 66 h at 37° C.

### HepG2 cell viability

Cell counting kit-8 assays (CCK-8, Dojindo, Tokyo, Japan) were used to determine the viability of HepG2 cells. HepG2 cells were plated onto 96-well plates at a density of 5 x 10^3^ cells per well and incubated overnight at 37° C. This was followed by incubation with oleate/palmitate for 0, 12, 24 and 36 h, after which the cells were incubated with 10 μL CCK-8 reagent for 2 h at 37° C. The optical density (OD) value of each well at the wavelength of 450 nm was then measured using a microplate reader (Thermo Fisher Scientific).

### Flow cytometry

Annexin V-FITC/PI Apoptosis detection kits (Thermo Fisher Scientific) were used to assess cell apoptosis. HepG2 cells were incubated with oleate/palmitate for 0, 12, 24 or 36 h and then fixed in 70% pre-cooled ethanol and washed twice with cold PBS. Thereafter, the cells were incubated with 5 μL of Annexin V-FITC and 5 μL of propidium iodide (PI) for 15 min at room temperature in the dark. Apoptotic cells were then detected using a flow cytometer on a BD FACSCalibur system (FACScan; BD Biosciences, Franklin Lake, NJ, USA).

### Measurement of intracellular ROS levels

ROS generation was measured using 29,79-dichlorodihydrofluorescein (DCFH-DA, Sigma Aldrich, St. Louis, MO, USA) according to the manufacturer’s protocol. HepG2 cells were incubated with oleate/palmitate for 24 h, after which they were washed twice with PBS and incubated with 10 μM DCFH-DA for 20 min at 37° C in the dark. The fluorescent signals were then detected using flow cytometry (BD Biosciences)

### Oil red O staining

Lipid deposition in HepG2 cells was assessed by staining with Oil red O as previously described [27]. HepG2 cells were fixed in 10% formalin for 5 min, after which the cells were washed with isopropanol, stained for 30 min with Oil Red O reagent (Sigma Aldrich), and counter-stained for 1 min with hematoxylin. The lipid deposition in cells was observed under a fluorescence microscope (Leica, Buffalo Grove, IL, USA). The Oil red O positive cell count was measured with the ImageJ 1.47 software.

### Detection of MMPs

JC-1 assay kits (Thermo Fisher Scientific) were used to evaluate mitochondrial depolarization. HepG2 cells were incubated with 1 mL of JC-1 staining solution for 20 min at 37° C in the dark. The stained cells were then washed twice with PBS and resuspended in PBS for analysis with a flow cytometer (BD Biosciences).

### Analysis of mitochondrial morphology

Changes in mitochondrial morphology were evaluated using transmission electron microscopy (TEM). HepG2 cells were incubated with oleate/palmitate (2:1 molar ratio) for 24 h. Subsequently, HepG2 cells were fixed by 2.5% glutaraldehyde (Sigma-Aldrich) in 0.1 M phosphate buffer for 2 h at 4° C and then centrifuged at 1000 rpm for 5 min. After that, HepG2 cells were fixed in 1% osmium tetroxide in 0.1 M phosphate buffer for 1 h. Later on, the fixed cells were dehydrated by a graded series of ethanol, and then embedded in epoxy resin. Finally, the ultrastructures of cells were captured using a TEM (H-600IV, Hitachi Ltd., Japan).

### Mitochondrial staining

HepG2 cells were incubated with 200 nM mito-tracker green (MTG) reagent (Beyotime, Beijing, China) for 30 min at 37° C. Cell nuclei were counterstained for 30 min with DAPI (Thermo Fisher Scientific). Mitochondria were observed under a confocal laser scanning microscope (Leica, Buffalo Grove, IL, USA).

### Western blotting

BCA protein assays (Thermo Fisher Scientific) were used to measure protein concentrations. Proteins were separated on 10% SDS-PAGE and then transferred onto a polyvinylidene fluoride membranes (PVDF, Millipore, Billerica, MA, USA). The membranes were then blocked with 5% skim milk for 1 h at room temperature before incubation overnight at 4° C with the primary antibodies, anti-GPX4 (1:1000, Abcam) and anti-GAPDH (1:1000, Abcam). The membranes were then incubated with horseradish peroxidase-conjugated goat anti-rabbit IgG secondary antibody (1:5000, Abcam) for 1 h at room temperature. The bands were visualized using an enhanced Chemiluminescence Detection System (Thermo Fisher Scientific) and quantified using ImageJ.

### Quantitative real-time reverse transcriptase-polymerase chain reaction (qRT-PCR)

Total RNA was extracted from cells with TRIzol reagent (Thermo Fisher Scientific), after which cDNA was synthesized using a SuperScript IV Reverse Transcriptase kit (Thermo Fisher Scientific)**.** For miR-188-3p determination, cDNA was synthesized using a PrimeScript RT reagent Kit (Promega, Madison, WI, USA). qRT-PCR was then carried out using a TaqMan Power SYBR Green PCR Mix kit (Thermo Fisher Scientific) on an Applied Biosystems 7500 Real Time PCR System (Applied Biosystems, Foster City, CA, USA). The PCR sequences were shown in Table 1. The level of hsa_circ_0048179 was normalized to the internal control GAPDH using the 2^−ΔΔCT^ method. The level of miR-188-3p was normalized to the internal control U6 using the 2^−ΔΔCT^ method.

**Table 1:** Primer sequences.

**Name**		**Primer sequences**
Hsa_circ_0048179	Forward	5’-CAGACCCGAAAATCCAGCG-3’
	Reverse	5’-CCCGGTACTTGTCCAGGTTA-3’
GAPDH	Forward	5’-TCAAGAAGGTGGTGAAGCAGG-3’
	Reverse	5’- TCAAAGGTGGAGGAGTGGGT-3’
Hsa-miR-188-3p	loop primer	5’GTCGTATCCAGTGCAGGGTCCGAGGTATTCGCACTGGATACGACTGCAAACC-3’
	Forward	5’- TGCGCCTCCCACATGCAGGGT-3’
U6	Forward	5’-CGCTTCGGCAGCACATATAC-3’
	Reverse	5’- AAATATGGAACGCTTCACGA-3’

### Luciferase reporter assays

HepG2 cells were used for dual-luciferase reporter assay. For the hsa_circ_0048179 reporter assay, wild-type (WT)-hsa_circ_0048179 or mutated-type (MT)-XIST plasmid were co-transfected with miR-188-3p agomir using Lipofectamine 2000 (Invitrogen, Carlsbad, CA, USA). For GXP4 reporter assays, WT-GXP4 or MT-GXP4 plasmid was co-transfected with miR-188-3p agomir using Lipofectamine 2000. Forty-eight hours later, the luciferase activities were detected using a Dual-Luciferase Reporter Assay System (Promega Madison, WI), with renilla luciferase activity serving as an endogenous control.

### FISH analysis

FISH analysis was performed as previously described [28]. The oligonucleotide probes were synthesized by Geneseed Biotech (Guangzhou, China). HepG2 cells were fixed for 30 min in 4% paraformaldehyde at room temperature, washed with distilled water, and then dehydrated through an ethanol series (70%, 90%, 100%). The air-dried cells were then incubated with FISH probes in hybridization buffer at 37° C overnight in the dark. Cell nuclei were counterstained for 30 min with DAPI (Thermo Fisher Scientific). Finally, images were captured using a FV10i confocal microscope (Olympus Corp, Tokyo, Japan).

### Statistical analysis

All experiments were performed in triplicate. Data are presented as the mean ± standard error (S.E.). Graphs were generated using GraphPad Prism software (version 7.0, La Jolla, CA, USA). One-way analysis of variance (ANOVA) and Tukey’s tests were carried out for multiple group comparisons. Values of P < 0.05 was considered statistically significant.

## Supplementary Material

Supplementary Figure 1
