# Supplementary material for: Hsa_circ_0048179 attenuates free fatty acid-induced steatosis via hsa_circ_0048179/miR-188-3p/GPX4 signaling
Source: Aging (Albany NY). 2020 Nov 18;12(23):23996–4008. doi: 10.18632/aging.104081 (PMC7762518; doi:10.18632/aging.104081)
Supplement: Supplementary Figure 1 [file aging-12-104081-s001.pdf]

SUPPLEMENTARY FIGURE

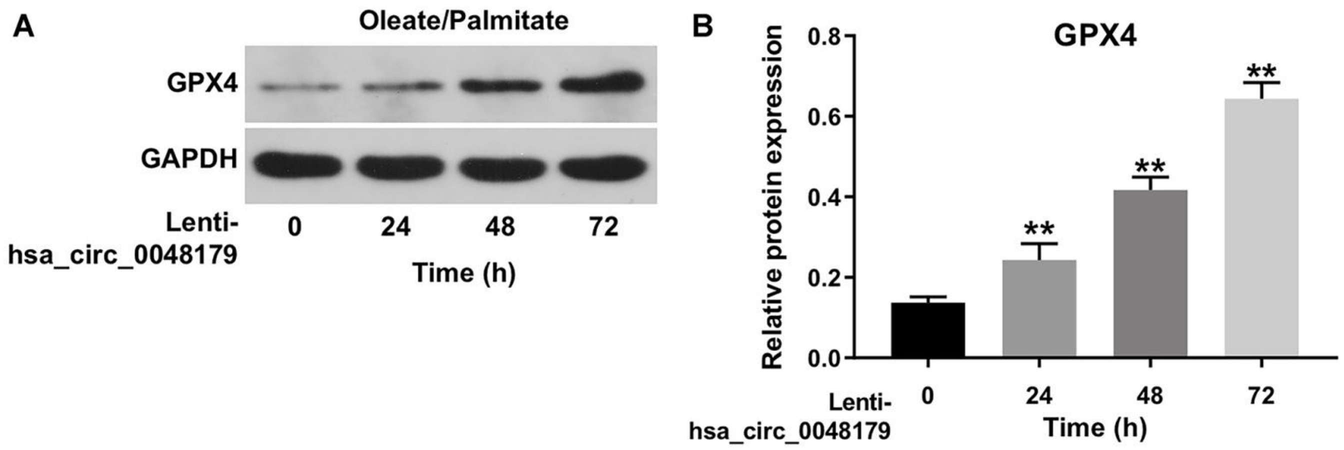

Supplementary Figure 1. Overexpression of hsa_circ_0048179 upregulated the expression of GPX4 in HepG2 cells. (**A**) HepG2 cells were transfected with hsa_circ_0048179 for 0, 24, 48 and 72 h, and then exposed to oleate/palmitate (2:1 molar ratio) for another 24 h. Levels of GPX4 expression in HepG2 cells were detected with western blotting. (**B**) Relative expression of GPX4 in HepG2 cells was quantified by normalization to GAPDH. **P<0.01 vs. control group.
